# A Theory-Based Self-Care Intervention with the Application of Health Literacy Strategies in Patients with High Blood Pressure and Limited Health Literacy: A Protocol Study

**DOI:** 10.1155/2018/4068538

**Published:** 2018-07-04

**Authors:** Homamodin Javadzade, Azam Larki, Rahim Tahmasebi, Mahnoush Reisi

**Affiliations:** ^1^Department of Health Education and Health Promotion, Bushehr University of Medical Sciences, Bushehr, Iran; ^2^Department of Biostatistics, Bushehr University of Medical Sciences, Bushehr, Iran

## Abstract

The purpose of this study is to assess the effectiveness of a theory-based self-care intervention with the application of health literacy strategies in patients with high blood pressure and limited health literacy. This is a randomized controlled trial, with measurements at baseline and 1 and 3 months follow-up. 100 patients with high blood pressure and limited health literacy will be randomly allocated to either an intervention group or a usual care control group. We will mainly establish the intervention model based on the principal health belief model components. Patients randomized to the intervention group will receive four educational sessions during four weeks. Considering the limited health literacy level of the patients of the study, health literacy strategies will be used in educational material design for enhancing the quality of the intervention. In order to cover these strategies, we will design four standard animated comics and fact sheets with illustrations and photos consistent with the health belief model constructs and educational sessions' topics. Data will be collected using some questionnaires and will be analyzed using the SPSS software. The findings of this study may assist with the development of a theoretical model for self-care intervention in patients with high blood pressure and limited health literacy.

## 1. Introduction

Hypertension is a chronic condition affecting approximately one billion individuals worldwide [1]. A 24% increase in developed and 80% in developing countries are predicted for the year 2025 and the increase is expected to be much higher than these predictions [2]. In this regard, it was reported to be 14 to 34 percent in Iran [3] and a systematically review study reported high rates of hypertension among males and females globally [4]. This chronic disease can lead to very serious consequences such as cardiovascular and kidney disease [5]. Due to the high prevalence of hypertension and its serious complications, the World Health Organization (WHO) assigned the theme of World Health Day 2013 to hypertension as a “silent killer, global public health crisis” [6].

Despite the benefits of evidence-based hypertension self-care behaviors in improving blood pressure, hypertensive patients usually have low compliance with the recommended self-care behaviors. Various studies all over the world suggest a high prevalence of uncontrolled blood pressure among people with hypertension [7, 8]. One approach that may advance blood pressure control and be practicable for the most of hypertensive patients is patient compliance with self-care behaviors [9]. The findings of a meta-analysis that examined the results of 87 studies indicated that optimal self-care in hypertensive patients could reduce systolic and diastolic blood pressure by 5 and 4.3 mmHg, respectively [10]. Self-care for people with high blood pressure includes a diet rich in fruits and vegetables, cessation of smoking, sufficient physical activity, antihypertensive medication, reduction in weight, saturated and total fat, and sodium, and moderate alcohol consumption [11]. Despite the benefits of evidence-based hypertension self-care behaviors in improving blood pressure, hypertensive patients generally do not follow medical or lifestyle recommendations and compliance with self-care behaviors in these patients is not desirable [12].

The WHO has emphasized patient education as an important strategy to improve the active participation of patients in their disease management process [13]. Self-care behaviors are influenced by demographic variables and modifiable psychological variables such as self-efficacy, perceived threats, perceived benefits and barriers [11, 14]. Although some studies have examined educational interventions in patients with high blood pressure, they often lack a theoretical framework describing how the educational emphasis on social, psychological, and cognitive variables can affect self-care behaviors in these patients.

Health Belief Model (HBM) is one of the most important theories of behavior change that have been widely considered in behavioral health sciences and successfully applied in the design of health interventions. This model has emphasized the role of moderating factors (demographic, social, and structural factors) and individual perceptions (perceived sensitivity, perceived severity, perceived benefits, perceived barriers, guidance for action, and self-efficacy) in determining the likelihood of performing a behavior [15]. According to this model, a person's decision and motivation to perform a particular behavior included items such as person's perception of being at risk (perceived susceptibility) and its seriousness (perceived severity), belief in the perceived action of usefulness to reduce the risk of disease, and understanding of the health benefits (perceived benefits) due to obstacles and moderating factors such as demographic and psychosocial variables (awareness) and people's judgments of their capabilities to execute given level of performance (self-efficacy)[16, 17]. The results of a cross-sectional study carried out in an earlier study showed that the HBM and its related structures, especially self-efficacy, perceived susceptibility, and severity of complications, are important determinants of self-care behaviors in hypertensive patients with limited health literacy [18].

Although education and especially the theory or model-based education are effective in controlling the blood pressure of patients, there is some evidence suggesting that limited health literacy of patients could act as a barrier in the result of the interventions [19]. Evidence suggests that limited health literacy patients may understand less than half of what is told to them during medical communication [20] Moreover, patients with low health literacy may be ashamed by their condition and hide their low level of literacy from healthcare providers who could possibly help [21]. In this regard, the results of a study on diabetic patients showed that despite the fact that 73% of patients with inadequate health literacy participated in diabetes education classes, 50% of them did not know the signs and symptoms of low blood sugar and 62% of them were unaware of the treatment methods for reducing blood glucose [22].

Choosing proper strategies, based on deeper understanding of needs and capabilities of patients with limited health literacy may help healthcare providers to communicate more efficiently with these patients [23]. Although low health literacy patients regularly rely on verbal instructions, they may have difficulty remembering and comprehending information [24]. Health literacy experts recommend incorporating a few effective and feasible strategies to advance communication and patients' understanding during the communication. Strategies such as plain language and using pictorial media in education will be used in this study.

Regarding high prevalence of hypertension in Iran and limited health literacy among a large population of these patients, it is indispensable to evaluate the effectiveness of an educational intervention based on influencing psychological factors using HBM applying health literacy strategies to promote self-care behaviors in patients with high blood pressure.

## 2. Material and Methods

### 2.1. Design

This randomized control trial (RCT) will be conducted with the high blood pressure patients attending to Haft-e-Tir comprehensive health services center of Bushehr, south of Iran. The trial was registered at the Iranian Registry of Clinical Trials with the IRCT code: IRCT2017011731999N1. The participants will be invited to the study to measure their health literacy level at first. Patients who have limited health literacy level according to S-TOFHLA result will be assigned to the two groups: HBM and health literacy strategies-based self-care intervention (trial) and usual care group (control).

### 2.2. Setting

All patients recruited are those diagnosed with high blood pressure who are referred to Haft-e-Tir comprehensive health services center in Bushehr city (south of Iran) between July 2017 and August 2017.

### 2.3. Patient Eligibility

The patients will be entered into the study if they are diagnosed with high blood pressure. Inclusion criteria for this study are as follows: (a) passing at least 6 months since the definitive diagnosis of the disease, (b) being able to read, write, and speak Persian, (c) being 30 years old and over, (d) having no severe complications caused by hypertension, including cardiovascular disease, kidney disease, retinal disease, and stroke, and (e) having the desire to participate in the study. Exclusion criteria for the study consist of (a) causing severe complications from hypertension, including cardiovascular disease, kidney disease, retina, and stroke during the study and (b) missing attending educational classes more than once.

### 2.4. Ethical Consideration

The study protocol will follow the principals of the “Declaration of Helsinki.” The participants will be told that they can withdraw from the study at any time and all information will be kept secret and anonymous. This study was approved by Bushehr University of Medical Sciences Ethics Committee (Number IR.BPUMS.REC.1395.128). Informed consent will be obtained from all the participants who will agree to participate in the study.

### 2.5. Sample Size

According to the study performed by Eftekhar Ardebili et al., 2014 [25], the effect size for the study has obtained based on 90% power, 5 % type one error level, and 1 unit difference between the mean score of knowledge before and, after the intervention, 40 patient per group have been estimated. Considering a dropout rate of 10% during the study, we aim to recruit 50 patients per group.

### 2.6. Measurement and Procedure

After checking the medical records file in the Haft-e-Tir comprehensive health services center and invitation of patients who meet the inclusion criteria for the study, invited participants will undergo a screening test by Test of Functional Health Literacy in Adults (S-TOFHLA) [26] for selecting those who have limited health literacy. S-TOFHLA is a valid and widely used measure which takes 12 minutes or less to administer. The S-TOFHLA includes reading comprehension and numeracy sections and uses actual materials that patients might encounter in the healthcare setting, such as medication label instructions. The sum of the 2 sections yields the S-TOFHLA score, which ranges from 0 to 100. Scores on the S-TOFHLA are classified and interpreted as follows: inadequate health literacy (scores 0 to 53): individuals will often misread the simplest materials, including prescription bottles and appointment slips and the instructions for an upper gastrointestinal tract radiograph series; marginal health literacy (scores 54 to 66): individuals perform better on the simplest tasks but have difficulty comprehending the Medicaid rights and responsibilities passage; adequate health literacy (scores 67 to 100): individuals will successfully complete most of the tasks required to function in the healthcare setting, although many still have difficulty comprehending more difficult information (i.e., materials written above a 10th grade reading level). In this study patients with both inadequate and marginal health literacy are considered limited health literacy. The valid Persian version of the scale shows adequate internal reliability for numeracy (Cronbach' s *α* =0.69) and for reading comprehension (Cronbach' s *α* = 0.78) [27].

Participants of the study will undergo three measurements: on entry to the study (pretest) and 1 and 3 months after having gone through the intervention for following up. Selected patients will go through the pretest and a then one-month intervention, 1 and 3 months after intervention follow-up.

### 2.7. Randomization and Blinding

From all hypertensive patients who had medical records in the Haft-e-Tir comprehensive health services center and were eligible for the study, we will select for the invitation by simple random sampling. Then we will screen them for health literacy stage. After completing the inform consent form, limited health literacy patients will randomly allocate to either the control or the intervention group. Randomization of the participants will be done using permuted block randomization. Due to the nature of this intervention, blinding of the patients to the allocation will not be completely possible.

### 2.8. Outcome Measures

**Figure 1:**
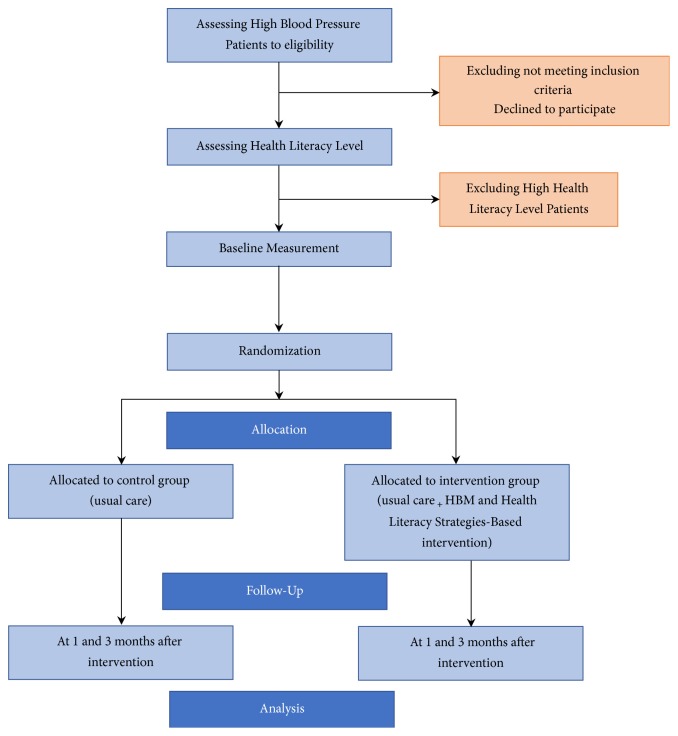
Study flow.

The outcome will be assessed at the baseline, one and 3-month after intervention [Figure 1]. Baseline measurement includes demographic (age, sex, marital status, education, job, income, smoking, and duration of the disease), HBM constructs questionnaire (perceived susceptibility, perceived severity, perceived barriers, perceived benefits, and self-efficacy), and self-care behaviors questionnaire.

### 2.9. Primary Outcome Measures

#### 2.9.1. Self-Care Behavior

Self-care behaviors will be determined using the 31-item hypertension self-care activity level effects (H-scale) prepared by Findlow [28]. This scale aims to help physicians for a better guidance to hypertensive patients who are looking for attaining blood pressure control [29]. The H-scale surveys the level of self-care by questioning about the number of days per week on which an individual carries out a self-care behavior. The H-scale was previously validated in Persian patients with high blood pressure [28]. The Persian version consisted of 27 items that measure the hypertension self-care activities with the following domains: medication adherence (3 items), physical activity (2 items), low-salt diet (10 items), smoking (2 items), alcohol (1 item), and weight management (9 items). The Persian version of the scale shows adequate internal consistency. Cronbach alphas were as follows: medication adherence (Cronbach's *α* = 0.91), low-salt diet (Cronbach's *α* = 0.72), physical activity (Cronbach's *α* = 0.96), smoking (Cronbach's *α*= 0.91), and weight management (Cronbach's *α* = 0.85).

### 2.10. Secondary Outcome

#### 2.10.1. Knowledge of Hypertension

 Hypertension knowledge will be assess by using Hypertension Knowledge Level Scale (HK-LS); this 22-item scale is prepared by Erkoc et al. [30]. Hypertension Knowledge Level Scale (HK-LS) will assess respondents' knowledge in defining hypertension, lifestyle, medical treatment, drug compliance, diet, and complications of hypertension. Each item is a full sentence that is either correct or incorrect. And each item is prepared as part of a standard answer (correct, incorrect, or do not know). Motlagh et al. have validated this questionnaire in Iranian population [28]. In Persian version in the validation process, two items were excluded from the scale and the final version has 19 true/false items.

#### 2.10.2. Health Belief Model Constructs

In order to assess the constructs of health belief model, a researcher made the questionnaire that will be used. Items developed for susceptibility, seriousness, benefits, barriers, and self-efficacy focused on self-care behaviors in hypertensive patients. 39 items with 5-point Likert answers will be used. Nine items for perceived benefits, 7 items for perceived barriers, 9 items for perceived susceptibility, 6 items for perceived severity, and 10 items for perceived self-efficacy were written. For determination of content validity, the list items were distributed to judges who were faculty members and PhD candidates and they were quite familiar with HBM constructs. In content validity, altering the format of questions and ignoring irrelevant questions were done. Then, mean Content Validity Index (CVI) and Content Validity Rate (CVR) of the questionnaire were calculated as 0.94 and 0.91, respectively. Reliability of the scale was calculated and the Cronbach's alpha values of knowledge questions were 0.71, 0.70, 0.70, 0.82, and 0.85 for perceived susceptibility, perceived severity, perceived barriers, perceived benefits, and perceived self-efficacy, respectively.

### 2.11. Intervention

#### 2.11.1. Theoretical Framework

HBM constructs are the guidelines for the educational intervention design in this study. In the HBM model, perceived severity, perceived benefits, perceived barriers, and perceived self-efficacy are the determinant components. We will mainly establish the intervention model based on the principal HBM components. Patients randomized to the intervention group will receive four groups of educational sessions (lasting 50-60 min) during four weeks. Topics of the first session are “what is high blood pressure?” and “what is self-care for hypertension?” This session will focus on the strategies to enhance perceived susceptibility and perceived severity of patients toward the disease. In this session, the patients will face the problem (risk of complications of hypertension problems) and they will be threatened (perceived susceptibility). Then, they will understand the depth of the risk and seriousness of complications (perceived severity).

In the second session, patients will learn and discuss healthy diet (such as low salt, low fat, fruit, and vegetable-rich diet). In the third session, patients will learn about physical activity and weight management. The fourth session will focus on medication adherence and avoidance of smoking and alcohol drinking. The importance of self-care behaviors, being physically active, adhering to medication, avoidance of smoking and alcohol drinking, and also possible barriers to a continuous healthy diet, will be discussed. In this session, brainstorming the possible and trained solutions for those barriers will cover the perceived barriers. By discussing the benefits and useful outcomes of adherence to the self-care behaviors, patients will believe the benefits and possibility of their behaviors (perceived benefits). Introducing an appropriate role model (strategy of vicarious experiences [Mrs. Karimi]) in video comics, providing solutions to address possible obstacles in removing the barriers, and giving verbal persuasions to positive expressions of the patients were used to enhance the self-efficacy of individuals for self-care behaviors.

Considering the limited health literacy level of the patients of the study, health literacy strategies will be used in educational material design for enhancing the quality of intervention. In order to cover these strategies, we will use four animated comics and fact sheets with a lot of illustrations and photos which are consistent with the HBM constructs and educational sessions' topics. Animated comics will be produced as media that will be shown to the intervention group patients at the beginning of each educational session to help them start discussing the topic and learn more about the self-care throughout the story of them.

In order to design these animated comics, the research team defines the characters of the story of a hypertensive patient (Mrs. Karimi), her family and friends and also her physician. The story of each part of the comic will cover the topic of the respective educational session. In the scenario of the animated comics we will make some recommendations for self-care behaviors to hypertensive patients. In the next step, the research team will design the storyboard of each part of the animated comic and then the characters and backgrounds will be illustrated compatible with the culture of the patients of the study using Adobe Illustrator software by a graphic designer. At last, the sounding and animating of each part of the storyboard will be done using Adobe After Effect software by an animation team.

The fact sheets that contain the most important recommendations and reminders of the educational topic will be designed based on existing standards and guidelines for designing simple and comprehensible educational media for limited health literacy patients, such as Simply-Put [31], which is provided by the Center for Disease Prevention and Control as a comprehensive guide for designing simple, understandable media. For optimal comprehension and compliance, it is suggested that patients' educational materials will be written at a sixth-grade or lower reading level, preferably including pictures and illustrations [32]. Therefore, Gunning Fog Index will be used to assess the readability of these written materials. This formula determines how difficult it is to read and understand a piece of writing. Readability will be calculated by the following formula: GFI = [(number of words/number of sentences) + number of “difficult words”] x 0.4 [33]. The number that results from the following calculation correlates with the grade level. All the sentences will design and modify in such a way that based on this indicator, scores less than 6 will be obtained for the readability level [32]. Each designed fact sheet will be given to the intervention group patients at the end of each educational session.

### 2.12. Statistical Analysis

Survey data will be coded and analyzed using the Statistical Package for the Social Sciences (SPSS) version 22. In addition to providing descriptive statistics, Chi-square test will be used to compare the distribution of qualitative demographical characteristics between the two groups before intervention. T-test will be used to compare the mean scores of knowledge and health belief model constructs (perceived sensitivity, severity perceived benefits, perceived barriers, and perceived self-efficacy) between the two groups. The Repeated Measure ANOVA analysis will be used to examine and compare changes in the mean scores of knowledge and constructs during the study period in each group and the comparison between the two groups. The post hoc analysis will perform within per groups to compare the times. The Kolmogorov–Smirnov test will be used for checking the normality distribution of the data. The P value of <0.05 will be considered statistically significant.

## 3. Discussion

The purpose of this study is to assess the effectiveness of a theory-based (HBM-based) self-care intervention by the use of health literacy strategies in limited health literacy hypertensive patients. To our knowledge, there is no published study investigating the use of behavioral change theories and health literacy strategies simultaneously to design an intervention to improve self-care in patients with high blood pressure. This protocol has the potential to improve the standard care of patients with limited health literacy by a simple, accessible, and relatively inexpensive way that could improve signs and symptoms. It is hoped that such educational intervention will produce more favorable health outcomes.

In this study the intervention will be based on health belief model. Applying this model would decrease methodological errors of the study which, in turn, would allow us to apply the valid and reliable educational strategies. Consequently, we are likely to believe that designing this educational program based on HBM constructs would be the key to the possible success. If it is found that the intervention improves self-care behaviors, future experimental works will be required to identify the most important component of HBM-based intervention for hypertensive patients. The results of this study will provide useful insights into the role that health literacy strategies, and health literacy level can play a role in improving self-care and awareness of complications in hypertensive patients with limited health literacy. If successful, the intervention may be adopted in other patient populations and RCT study designs.

The proposed study has a number of potential limitations. First, there is only a limited time in which the participants can be recruited. Second, due to the nature of this intervention, blinding of the patients to the allocation is not completely possible in this study. Third, using self-report instruments for measuring self-care behaviors and adherence limits the ability to identify changes objectively. The fourth limitation is the use of questionnaires that their construct validity was not confirmed due to time limitation. However, appropriate content validity and reliability of the HBM questionnaires were properly met. Further studies should be done to measure long-term effects of the intervention as well as clinical outcomes.
